# *De motu cordis*: the future of regenerative medicine

**DOI:** 10.1007/s12471-018-1222-1

**Published:** 2019-01-08

**Authors:** A. Mathur

**Affiliations:** 1 Centre of Clinical Pharmacology, William Harvey Research Institute, Barts & The London Medical School, Queen Mary University, London, UK; 2 Barts NIHR Biomedical Research Centre, Barts & The London Medical School, Queen Mary University, London, UK; 3 Barts Interventional Group, Interventional Cardiology, Barts Heart Centre, St. Bartholomew’s Hospital, London, UK

In this edition of the *Netherlands Heart Journal* Mann et al. [1] describe the results of a clinical trial that was designed to address whether autologous bone marrow-derived mononuclear cells injected by the intramyocardial route could benefit patients with heart failure. The study also investigated whether any benefits were related to patients in whom ischaemia was demonstrated (suggestive of viable myocardium) as opposed to scar. The study failed to achieve its primary end-point and has been reported as a negative study given that none of the clinical parameters appeared to improve in the cell-treated patients compared to the controls.

In the current climate of scepticism relating to cell-based therapies as treatment for heart disease it is all too easy to accept this as another piece of evidence suggesting that the autologous cell-based therapy approach has failed after many years of small phase II clinical trials showing mixed results but no clear-cut overall efficacy signal. However before closing the door on this promising area of research (which provides a relatively cheap way of delivering cell-based therapies) we need to understand why others have shown beneficial effects of the autologous approach and whether further work is needed before reaching a definitive conclusion.

It should be remembered that chronic heart failure represents an unmet need with regard to new therapies that can change symptomatic and prognostic outcome. The number of patients diagnosed with heart failure is increasing across Europe despite advances in pharmacological, device-related and pathway-specific changes to the management of heart failure. Heart transplantation continues at a low level across Europe and is not sufficiently available to treat this increasing burden of disease. Mechanical support devices have undergone substantial refinement but still provide a high cost option to the selected few.

It is therefore important to carefully consider any new therapies that may help solve the heart failure burden as well as being ready to dismiss novel approaches following a rigorous programme of evaluation.

Regarding the use of autologous cells for the treatment of heart failure the results of the metanalyses suggest a beneficial effect. However this has not been taken forward in the design of a definitive large trial to answer the question in the usual recognised approach. Instead small-scale trials with differing methodologies continue to be performed, adding to the confusion. Therefore there remains a need to ensure that clinical trials are performed with rigour and attention to detail. In the same way that the culturing of cells relies on meticulous attention to reagents and culture conditions, cell therapy in the clinic should be considered similarly. Previously the European Society of Cardiology Task Force for Stem Cells in Cardiac Disease published recommendations [2] regarding the need to rationalise clinical trials in this field to avoid the accumulation of trial data with varying methodologies and hence the danger of creating a large pool of unrelated knowledge in which a clear signal would be difficult to detect. Unfortunately the complexities of cell biology combined with a failure to standardise the methodology has meant that the field now has a collection of small trials with varying results and no obvious way forward. The BAMI phase III clinical trial [3] is one of the few examples in which an attempt has been made to design and complete a definitive trial (including standardisation of methodology) that is the result of a programme of work over nearly two decades that started with preclinical ‘discovery’ and ‘confirmatory’ studies that then moved to phase IIa and IIb clinical trials before the move to phase III. The results of the BAMI trial (autologous cell therapy in acute myocardial infarction) will be available in a few years’ time. Not only does a standardisation of the methodology in this field of research mean that data can be pooled, it also provides a way forward regarding regulatory approval should the trials show beneficial results. Standardisation is therefore a key point. Whilst small phase II trials are important to try and establish which method (patient selection, cell type, cell delivery etc.) to standardise, the need to lock the methodology as well as choose appropriate end-points still exists. As yet this has not happened in a meaningful way across the field.

So where does the trial reported by Mann et al. [1] fit? The trial highlights the realities of working in the field of translational medicine particularly in the context of cell therapies. From an academic perspective these trials are expensive and in order to decrease variability, defining strict inclusion criteria can lead to difficulty in trial recruitment which in time may lead to early termination of the trial for practical reasons as was the case in this study. The trial therefore failed to recruit to its primary statistical end-point and was not powered around an interim analysis. Drawing meaningful conclusions based on the statistical plan becomes difficult. However the data still should be informative in trying to progress the field. The researchers failed to demonstrate any trend to superiority in the cell-treated patients above those receiving placebo and are therefore drawn to the conclusion that the intramyocardial approach is not effective. This finding goes against the metanalysis previously published and the handful of small studies that did demonstrate a positive effect on cardiac function using autologous cells delivered by the intramyocardial route as opposed to intracoronary which was directly tested in the REGENERATE IHD trial [4]. This made intuitive sense versus the intracoronary approach based on the fact that the majority of patients with heart failure due to ischaemic heart disease have chronically occluded coronaries and thereby a lack of suitable conduits to supply the myocardium with injected cells (the role of the collateral circulation is unknown). Other small phase II studies have previously shown an efficacy signal using the intramyocardial approach although conversely the relatively large CCTRN trial [5] in the United States failed to meet its primary end-point using intramyocardial injection. Several explanations have been put forward to explain this discrepancy, including the use of automated instead of manual cell processing in the US study with a potential loss of ‘active cellular’ component.

Thus the rationale for intramyocardial delivery of autologous cell therapy for the treatment of heart failure is scientifically justified but not clinically proven. The trials that have been performed to date, including the work of Mann et al. [1], highlight the complex issues of developing biological therapies that have not been faced by established pharmacological and device-based treatments. Taken pragmatically the learning from these trials suggests that the logistics and cost of intramyocardial delivery of autologous or indeed any cell type will need a large effect size to justify this approach. Autologous cells themselves are difficult to commercialise and thus the allogenic cell may provide a more feasible approach. The main advantage of the allogenic or engineered cell is in the ability to generate intellectual property which in itself is crucial to raise the funding necessary for these expensive trials. The academic-driven approach applied to the autologous cell (particularly when unfractionated and without intellectual property) may well produce a cost-efficient treatment. However it also flawed based on the inability to attract significant financial resource—without which academia will fail to complete the rigorous approach that is needed for autologous cell therapy in ischaemic heart failure (cf. BAMI). Hence this line of clinical translation will disappear not through lack of scientific endeavour but through lack of commercial opportunity to deliver into the existing healthcare systems.

Taking all of this together the field of regenerative medicine as applied to the heart needs a rethink. Conceptually the ideas are solid. However the approach up until now has failed to live up to the high expectations of the cardiology community across Europe. New approaches to funding academic consortia are needed if we are to complete the scientific processes that are necessary to fully answer the questions raised by the phase II trials.

In order to progress the field, the European Society of Cardiology has recently established a Working Group on Cardiovascular Regenerative and Reparative Medicine—the aim of which is to develop the field beyond the current hiatus that now exists. We eagerly await the guidance from this group and the release of definitive results that will help decide which areas of regenerative medicine should be pursued and importantly what should be dropped.
